# Croatia needs a registry of patients undergoing direct current cardioversion for persistent atrial fibrillation/flutter

**DOI:** 10.3325/cmj.2016.57.403

**Published:** 2016-08

**Authors:** Mia Dubravčić, Petra Cukon, Siniša Car, Davor Puljević, Vladimir Trkulja

**Affiliations:** 1 School of Medicine, University of Zagreb, Zagreb, Croatia; 2 Department of Cardiology, General Hospital Varaždin, Varaždin, Croatia; 3 Department of Cardiology, University Hospital Centre Zagreb, Zagreb, Croatia

To the editor:

Direct current cardioversion (DCC) is a procedure used to restore sinus rhythm in patients with persistent atrial fibrillation (AF) or atrial flutter (AFL) (1). It is associated with a 1%-2% risk of thromboembolic events (2). In patients with persistent AF/AFL lasting longer than 48 hours, this risk can be reduced by adequate anticoagulation or exclusion of atrial thrombi using transesophageal echocardiography (TEE). Guidelines for the management of atrial fibrillation of the European Society of Cardiology (ESC) (2) suggest two approaches: a) prophylactic anticoagulation over at least 3 weeks before and 4 weeks after the procedure, implying that the international normalized ratio (INR) is between 2.0 and 3.0 if vitamin K antagonists (VKA) are used, or by using direct orally active anticoagulants (DOACs) (2-5); b) TEE with anticoagulation therapy immediately before (low molecular weight heparins with VKA) and over 4 weeks after the procedure (VKA with INR 2.0-3.0, or DOACs) (2).

The aim of this study was to retrospectively assess compliance of the management of patients with persistent AF/AFL lasting longer than 48 hours at one university hospital (UH) and one general hospital (GH), considered representative of tertiary and secondary health care providers in Croatia, with the ESC guidelines. We identified all patients undergoing the procedure between September 3, 2010 and September 3, 2014 at UH and between March 1, 2009 and July 7, 2014 at GH. Patients were identified and confirmed through the hospital electronic databases and hardcopy archives.

**Figure 1:**
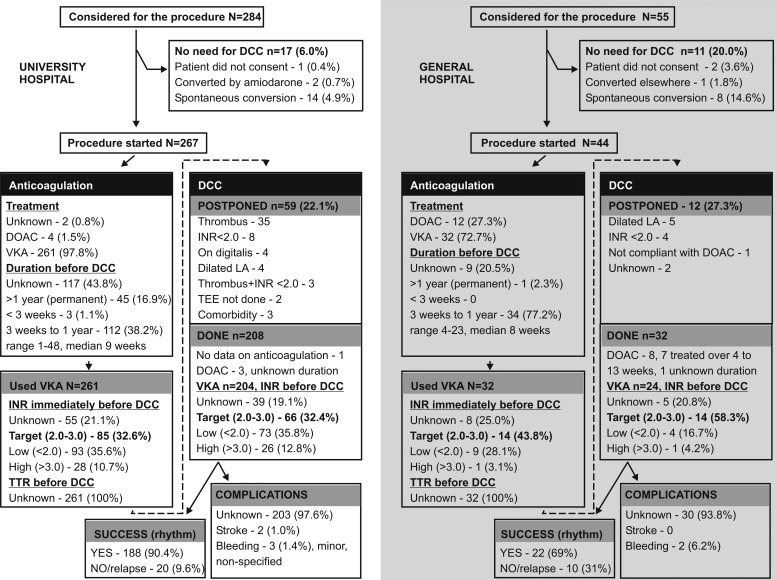
Flow diagram of patients considered for direct current cardioversion (DCC) due to atrial fibrillation/atrial flutter identified in the present study at two hospitals (one university hospital, one general hospital) in Croatia, between 2009 and 2014. The steps of the procedures are indicated by the arrows. DOAC – direct orally active anticoagulant; INR – international normalized ratio; LA – left atrium; TEE – transesophageal echocardiography; TTR – time in therapeutic range; VKA – vitamin K antagonists.

339 patients (age 20 to 92 years, median 65, interquartile range 58-71; 67.9% men; 41.6% suffering the first AF/AFL episode), 284 at UH and 55 at GH, were initially considered for the procedure, but some were eventually dropped mainly due to spontaneous cardioversion to sinus rhythm (Figure 1). Considering those who entered the procedure, two main observations were made: a) at GH, the approach was compliant with the ESC guideline option based on prophylactic anticoagulation (over at least 3 weeks before DCC) for all, whereas at UH the approach was a combination of such an approach and the one based on the use of TEE (Figure 1); b) all records were strikingly sparse. For example, VKA were used for anticoagulation in the vast majority of the patients (97.8% at UH, 72.7% at GH), but no INR values were available for estimation of the time in therapeutic range (TTR) before DCC, and INR values on the day of DCC were missing for 21.1% patients at UH and 25.0% patients at GH (Figure 1). Moreover, no trace of a “structured” post-DCC monitoring/follow-up was found.

Considering those anticoagulated with VKA (n = 261 at UH, n = 32 at GH) (Figure 1), on the day of the planned DCC, the explicit evidence of an INR within the target range could be ascertained for only 32.6% patients at UH and 43.8% at GH (Figure 1), whereas INR was too low in 35.6% and 28.1% of the patients, respectively, and too high in 10.7% and 3.1% of the patients, respectively (Figure 1). The planned DCC was postponed for various reasons in 22.1% patients at UH and in 27.3% patients at GH (Figure 1). Considering those who actually underwent DCC and were anticoagulated by VKA (n = 204 at UH, n = 24 at GH), the explicit evidence of an INR within the target range at DCC was found for only 32.4% of the patients at UH and for 58.3% at GH, whereas INR was too low in 35.8% and 16.7% of the patients, respectively, and too high in 12.8% and 4.2% of the patients, respectively – no data were available for the remaining patients (Figure 1). DCC was successful in 90.4% patients at UH and in 69.0% at GH (Figure 1), but no records on possible complications (or explicit records of a lack of complications) could be identified for 97.6% patients at UH and 93.8% patients at GH (Figure 1). Yet, despite the lack of any trace of a safety follow-up, 2 (1.0%) patients treated at UH were hospitalized at UH within the subsequent 14 days due to a stroke (no cases at GH), and 1.4% at UH and 6.2% at GH experienced bleedings (Figure 1). Moreover, 2 more patients at UH experienced a stroke, but DCC was actually postponed due to a thrombus in the left atrium visible by TEE. One of them had INR<2.0 (on the day of the planned DCC), and for the other, the INR value was unknown.

In patients with AF/AFL, thromboprophylaxis is an essential treatment as it greatly reduces the risk of stroke, and if VKA are used for the purpose, they largely fail to achieve the therapeutic objective if time with INR in the therapeutic range is below 60% (6-8). DCC further increases the risk of thromboembolic events, hence thromboprophylaxis is a standard preparatory procedure (2). Since DCC is not a very common procedure, evidence about efficacy/safety of thromboprophylactic treatments comes mostly from subgroup analyses in large clinical trials or from observational studies (5). Under such circumstances, systematic follow-up of patients in daily practice, eg, through registries/structured databases at the institutional or national levels (9,10) could provide sound grounds for efficacy and safety assessment, identification of modifying factors and, if needed, adjustment of practice. The present data clearly demonstrate that such a re-appraisal in the two observed institutions – which set the indication for DCC, determine the overall protocol, and execute it – would be impossible due to extremely sparse documentation inappropriate for any reliable assessment. Moreover, the available data strongly suggest that the thromboprophylactic practice and follow-up is poor. Overall, the present work emphasizes a need for establishment of a registry of patients with AF/AFL undergoing DCC at the national level.
